# Scorpion venom peptides: Novel therapeutic approaches for inflammatory and hepatic disorders

**DOI:** 10.1016/j.iliver.2026.100255

**Published:** 2026-08-18

**Authors:** Jwal Doctor, Zeal Parikh, Advaita Chauhan, Anirudh Raina

**Affiliations:** a Arnold & Marie Schwartz College of Pharmacy and Health Sciences, Long Island University, New York, NY, 11201, USA; b Faculty of Pharmacy, The Maharaja Sayajirao University of Baroda, Vadodara, 390001, India

**Keywords:** Scorpion venom peptides, Hepatic disorders, Anti-inflammatory effects, Genomics, Transcriptomics

## Abstract

Chronic hepatic disorders, such as metabolic dysfunction associated steatohepatitis (MASH), alcohol associated liver disease (ALD), and viral hepatitis (Hepatitis B virus [HBV]/Hepatitis C virus [HCV]), are primarily driven by persistent immune-mediated inflammation and hepatic stellate cell activation leading to fibrosis, yet conventional therapies lack tissue and molecular specificity. Scorpion venom peptides, refined through evolutionary selection, provide highly potent, target specific scaffolds capable of modulating intrahepatic inflammatory networks. Recent *in vivo* preclinical studies indicate that voltage gated potassium (Kv1.3) channel blocking peptides, such as BmKK2, significantly reduce macrophage activation and inhibit downstream cytokine production, effectively ameliorating diet-induced steatohepatitis and tissue scarring in murine models. Engineered hepatotropic candidates, such as Smp76 and Mucroporin-M1, demonstrate dual therapeutic functions: they neutralize extracellular Hepatitis C particles and suppress key host transcription factors necessary for Hepatitis B replication. This review systematically examines scorpion venom peptides organized by disease category, covering their historical development, structural classification into disulfide-bridged and non-disulfide-bridged families, ion channel specificity, hepatic anti-inflammatory and antiviral mechanisms, and translational challenges including nano-formulation delivery strategies and computational drug design. These target-specific peptides are ultimately positioned as promising molecular leads that may bridge targeted immunomodulation with the resolution of chronic, progressive liver injury.

## 1 Introduction

Scorpions represent one of the oldest surviving arachnid lineages, with an evolutionary history extending approximately 400 million years that has contributed to their broad geographic distribution and extensive species diversity. Currently, more than 2,700 extant scorpion species, representing 20 families, have been documented. Beyond their chemical, toxic, pharmacokinetic, and pharmacodynamic properties, scorpion venom has significant medical relevance. Scorpion venom is a complex biological mixture of proteins, peptides, enzymes, and non-peptide small molecules. For centuries, dried scorpion venom preparations have been incorporated into traditional Chinese medicinal practices for applications including diuresis, cardiovascular support, and anesthesia.^1^ While traditional use drew broadly on this mixture, modern research has established the peptide fraction as the principal determinant of pharmacological activity.^2^ Among these components, venom peptides frequently act as ligands that selectively interact with receptors involved in inflammatory processes.^2^

For instance, venom peptides can selectively target specific ion channels, offering the potential to modulate neuronal signaling pathways associated with pain and inflammation.^3^ Certain venom components can also interact with receptors that activate immune cells, subsequently triggering cytokine generation, which leads to inflammation. By selectively blocking ion channels linked to neuronal signaling, venom peptides hold promise for managing pain and inflammation more effectively than traditional NSAIDs.^4^ This specificity is thought to result from evolutionary optimization, as nature has “fine-tuned” these peptides to maximize their efficacy while minimizing the energetic cost to the organism that produces them. This characteristic suggests the potential for therapies that provide precise inflammatory control with fewer side effects compared to conventional drugs.^5^^,^^6^

Peptides and proteins derived from scorpion venom exhibit significant potential as anti-inflammatory agents. However, their pharmaceutical application is hindered by the complex processes involved in their isolation and purification. These technical challenges have limited the identification of therapeutic molecules to only a small fraction of the total potential. Nevertheless, recent advancements in genomics, transcriptomics, and computational techniques have enhanced our understanding of the structure and function of venom peptides.^7^ Substantial preclinical research remains necessary to elucidate their mechanisms of action, pharmacokinetics, and toxicity before their development as viable therapeutic options.

## 2 Historical evolution of scorpion venom therapeutics

The evolution of scorpion venom from an ancient empirical remedy to a contemporary molecular discipline exemplifies a broader transformation in the field of natural product drug discovery. Historically, traditional medical systems, particularly traditional Chinese medicine (TCM), have utilized dried whole body scorpion tissues or crude venom extracts in a rudimentary manner. The scorpion Buthus martensii Karsch (BmK), referred to in TCM as “Quan Xie”, has been documented in the *Shu Bencao* from the Later Shu period (935–960 AD.) and has been used for over a millennium to address conditions such as epilepsy, stroke, rheumatism, and chronic pain^8^^,^^9^. These early therapeutic practices lacked molecular specificity and often involved a trade-off between therapeutic efficacy and the physiological risks associated with systemic envenomation.

The field of scorpion toxinology began to evolve significantly during the mid-to-late 20th century, largely due to advancements in protein separation technologies, including size-exclusion chromatography and high performance liquid chromatography (HPLC). These methodologies enabled researchers to effectively separate toxic, high molecular weight macromolecular enzymes from smaller bioactive peptide fractions. This separation demonstrated that the therapeutic potential of the venom is primarily located within these lower molecular weight sequences, which can be isolated without the dense, toxic matrix of the crude venom.^10^

At the onset of the 21st century, the field underwent significant advancement due to the incorporation of high throughput “omics” technologies. The transcriptomic and genomic sequencing of scorpion venom glands addressed the longstanding issue of limited physical venom availability by enabling the direct extraction, amplification, and documentation of complete peptide precursor sequences. Concurrently, progress in high resolution structural biology, including NMR spectroscopy and X-ray crystallography, clarified the highly conserved structural frameworks, such as specific disulfide arrangements, that protect these peptides from rapid proteolytic degradation within host systems.^10^^,^^11^

In recent developments, the field has integrated computational medicinal chemistry, molecular docking, and molecular dynamics (MD) simulations. These sophisticated structural bioinformatics tools facilitate the modeling, prediction, and engineering of interactions between synthetic venom peptide analogs and human disease receptors. This transition from descriptive toxicology to predictive, structure-based peptide design now underpins scorpion-derived drug discovery and aids in targeted clinical applications.^11^^,^^12^

## 3 Chemical composition and ion channel classification of scorpion venom peptides

Scorpion venom is a complex biological mixture shaped by evolutionary pressure. The complete crude matrix contains lipids, salts, nucleotides, low molecular weight biogenic amines, high molecular weight metabolic enzymes, and small neurotoxic proteins.^13^ From a biomedical and pharmacological perspective, the most significant components are small peptides under 10 kDa. These fragments have evolved to target and modulate voltage-gated ion channels within the central and peripheral nervous systems, making them the primary focus of modern anti-inflammatory bioprospecting.^7^^,^^14^^,^^15^

Based on their core structural conformations and the presence of internal stabilizing bonds, scorpion venom peptides are divided into two primary structural superfamilies: disulfide-bridged peptides (DBPs) and non-disulfide-bridged peptides (NDBPs).^3^

### 3.1 Disulfide-bridged peptides (DBPs)

DBPs represent the most broadly characterized class of scorpion venom components. They are structurally defined by a rigid molecular basis held together by three to four highly conserved internal disulfide bonds. These covalent cross-links provide excellent thermal, chemical, and proteolytic stability, preserving the peptide's active 3D conformation under complex physiological conditions.^3^^,^^13^ Functionally, DBPs act as highly selective, potent modifiers or blockers of voltage-gated ion channels permeable to sodium (Na^+^), potassium (K^+^), calcium (Ca^2+^), and chloride (Cl^−^) ions. Because ion channels govern cellular excitability, transmembrane signaling, and immune cell proliferation, DBPs display broad therapeutic actions. These include selective immunomodulation, antiproliferative effects against human malignancies, and targeted blockade of voltage-gated potassium (Kv1.3) channels on chronically activated effector memory T cells, which effectively halts downstream autoimmune responses.^11^^,^^16^

### 3.2 Non-disulfide bridged peptides (NDBPs)

In contrast to DBPs, NDBPs lack internal cysteine cross-links, presenting instead as short, linear, and often amphipathic structures. Because they lack rigid internal structural reinforcement, linear NDBPs are more conformationally flexible in solution, frequently adopting defined α-helical geometries only upon direct contact with target lipid membranes.^3^ Rather than solely occluding physical ion channel pores, NDBPs often interact directly with the lipid bilayer of cells or target cell surface pattern recognition receptors, such as Toll-like receptor 4 (TLR4). This interaction allows them to modulate intracellular inflammatory signaling cascades, disrupt bacterial or viral membranes, and balance systemic pro-versus anti-inflammatory cytokine profiles without causing off-target neural toxicity.^11^^,^^17^ Integrating these structural classes with their functional targets provides a unified framework for understanding how these peptides achieve their therapeutic effects.

## 4 Scorpion venom peptides organized by major disease categories

### 4.1 Autoimmune and chronic inflammatory disorders

Chronic autoimmune disorders are characterized by aberrant immune activation and uncontrolled cytokine release. Voltage-gated Kv1.3 potassium channels are highly expressed on chronically activated effector memory T cells, making them an important therapeutic target for autoimmune diseases.^18^^,^^19^ When venom-derived DBP blockers selectively occlude the Kv1.3 channel pore, they disrupt the intracellular calcium signaling required for T cell activation, arresting downstream cytokine production and mitigating autoimmune progression.

The primary archetypal blocker of this pathway is BmKK2, a highly thermostable DBP isolated from Buthus martensii Karsch. BmKK2 selectively blocks the Kv1.3 channel pore on macrophages and T cells, downregulating the intracellular TLR4/NF-κB (Nuclear factor kappa-light-chain-enhancer of activated B cells) pathway and halting downstream NLRP3 inflammasome activation. This targeted suppression prevents the cleavage and release of potent proinflammatory cytokines such as TNF-α and IL-1β.^20^

**Fig. 1:**
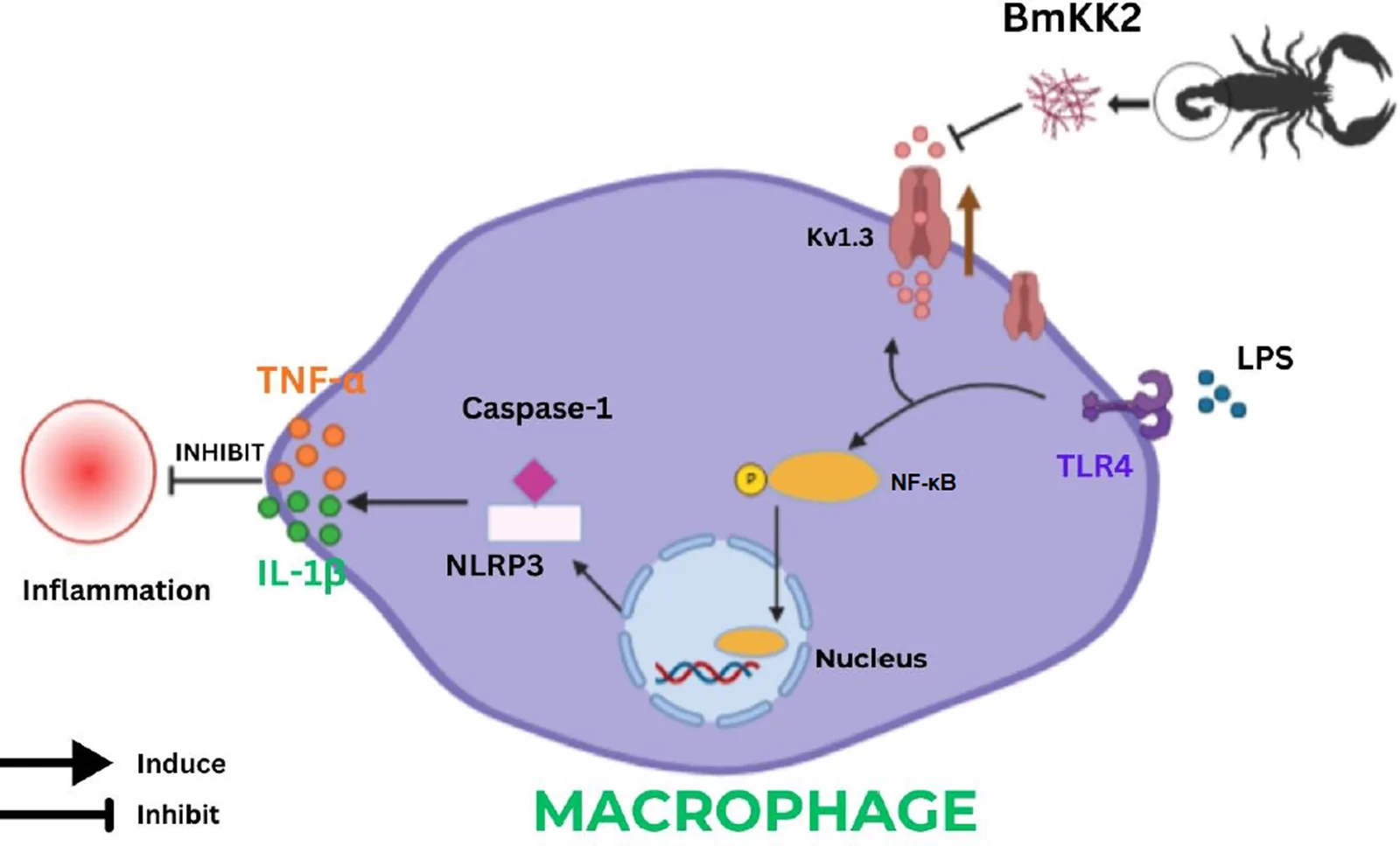
Immunomodulatory mechanisms of scorpion venom-derived peptides. the coordinated suppression of cytokine release and T-cell activation achieved by scorpion venom-derived peptides, offering a multi-pronged approach to autoimmune and inflammatory pathology in animal models. Adapted from Ref. 20, Copyright © 2023 Elsevier. Abbreviations: BmKK2, (*Buthus martensii* Karsch potassium channel toxin 2); IL-1β, (Interleukin-1 beta); Kv1.3, (Voltage-gated potassium channel 1.3); LPS, (Lipopolysaccharide); NF-κB, (Nuclear factor kappa-light-chain-enhancer of activated B cells); NLRP3, (family pyrin domain containing 3); TLR4,(Toll-like receptor 4); TNF-α, Tumor necrosis factor alpha.

Similarly, the toxin BmKTX (from M. martensii) and its engineered synthetic analogs (ADWX-1, BmKTX-D33H, BmKTX-19, and BmKTX-196) exhibit nanomolar affinity for the Kv1.3 channel, serving as specialized candidates for T cell-mediated autoimmune pathology.^21^ Beyond the Buthus genus, Vm24 (from Vaejovis mexicanus) achieves potent Kv1.3 inhibition without reducing overall T cell viability, selectively downregulating critical activation markers CD25 and CD40L.^18^^,^^19^. Additionally, hetlaxin, a DBP isolated from the Vietnamese forest scorpion Heterometrus laoticus, binds to Kv1.3 channels to produce a systemic anti-inflammatory effect that matches or exceeds the efficacy of the traditional NSAID ketoprofen,^22^ as illustrated in Fig. 1.

Complementing these channel-blocking DBPs are linear NDBPs isolated from the Tityus genus. Peptides ToAP3 and ToAP4 (from Tityus obscurus) target surface costimulatory molecules rather than ion channels. ToAP4 systematically downregulates the expression of major histocompatibility complex Class II (MHC-II) on bone marrow-derived dendritic cells (BMDCs), while ToAP3 downregulates essential costimulatory ligands CD80 and CD86. Working together, these peptides reduce TNF-α and IL-1β production at both the transcriptional and translational levels while upregulating the systemic anti-inflammatory cytokine IL-10.^17^ Furthermore, stigmurin (from Tityus stigmurus) and TsAP-2 (from Tityus serrulatus) attenuate systemic leukocyte migration and balance pro-versus anti-inflammatory profiles by modulating IL-4, IL-6, and IL-13 levels.^34^

### 4.2 Neurological inflammation and pain management

Neurological inflammation and nociceptive signaling represent interrelated pathways driven by localized ion channel hyperactivity and microglial activation. Venom peptides have evolved to modulate these channels, offering precise strategies for pain management and neuroprotection.^12^

The intracellular NF-κB and mitogen-activated protein kinase (MAPK) pathways are central drivers of this neuroinflammatory cascade. The NF-κB cascade is activated when IκBα (Nuclear factor of kappa light polypeptide gene enhancer in B-cells inhibitor, alpha) is degraded and the p65 subunit translocates to the nucleus, while phosphorylation of key MAPK components (ERK, JNK, and p38) amplifies the inflammatory signal. Disruption of this signaling axis in macrophages and microglia can halt the downstream production of neuroinflammatory mediators, including nitric oxide (NO), TNF-α, IL-6, and IL-1β.^12^

For direct nociceptive management, BotAF, a long chain peptide isolated from Buthus occitanus tunetanus, acts as a highly potent analgesic agent in rodent models.^35^ Similarly, Sc20 and St20 (from Scorpiops tibetanus) function as powerful immunosuppressive and analgesic peptides, altering systemic IFN-γ and TNF-α concentrations to alleviate delayed type hypersensitivity pain^36^.

To target neuroinflammation within the brain, scorpion venom heat resistant peptide (SVHRP), a component extracted from BmK crude venom, crosses the blood-brain barrier to attenuate microglial hyperactivation. Because cerebral ischemia-reperfusion injury and chronic epilepsy are driven by localized neuroinflammatory cascades, SVHRP's ability to suppress microglial reactivity significantly lowers seizure susceptibility and limits ischemic brain tissue damage.^37^

Additionally, the peptide Ts14 (from T. serrulatus) modulates structural tissue repair. *In vivo* sponge implant models show that Ts14 regulates fibrovascular tissue development, promotes localized angiogenesis, and reduces excessive extracellular matrix deposition. This regulation is mediated by suppressing myeloperoxidase (MPO) and N-acetyl-β-D-glucosaminidase (NAG) enzyme activities, which decreases excessive neutrophil infiltration and encourages healing macrophage phenotypes.^38^ Additional peptides having varying properties are listed in Table 1.

**Table 1:** Scorpion venom-derived peptides as therapeutic agents for inflammation.

Peptide Name	Scorpion Species	Ion Channel Target	Ref
BmK AS	B. martensii Karsch	Nav 1.3	[16]
BmK-YA	B. martensii Karsch	Mammalian opioid receptors	[23]
(BmK) AngM1	B. martensii Karsch	Nav, Kv	[24]
BmK AGAP	B. martensii Karsch	Nav 1.7	[25],[26]
ANEP	B. martensii Karsch	Nav 1.7	[27]
BmK AGPSYPU1	B. martensii Karsch	—	[28]
BmK AGPSYPU2	B. martensii Karsch	—	[29]
BmKM9 (rBmKM9)	B. martensii Karsch	Nav1.4, Nav1.5, Nav1.7	[30]
Amm VIII	A. mauretanicus	Nav 1.2	[31]
TsNTxP	T. serrulatus	Nav	[32]
Hetlaxin	H. laoticus	Kv1.3	[22]
Leptucin	H. lepturus	—	[33]
DKKSP1/2	B. martensii Karsch	Nav 1.8	[26]

## 5 Scorpion venom peptides in hepatic disorders

### 5.1 Therapeutic modulation of nonviral hepatic inflammation and fibrosis

While whole crude scorpion venom is known to induce sublethal hepatotoxicity upon systemic envenomation (characterized by transient hepatocyte swelling, granular degeneration, and elevated serum biomarkers including ALT, AST, ALP, and LDH^39^), the isolation and structural optimization of specific peptide fractions offer highly targeted therapeutic mechanisms within the liver.

Chronic nonviral hepatic disorders, including metabolic dysfunction-associated steatohepatitis (MASH), alcohol-associated liver disease (ALD), drug-induced liver injury (DILI), and progressive cirrhosis, are fundamentally driven by chronic, unmitigated inflammation. At the cellular level, this pathology is mediated by a complex crosstalk between resident hepatic macrophages (Kupffer cells), infiltrating bloodborne leukocytes, and quiescent hepatic stellate cells (HSCs).^40^^,^^41^

Direct preclinical evidence now supports the therapeutic relevance of scorpion venom peptides in this hepatic context. Xu et al. (2025) demonstrated that processed BmK scorpion venom gland aqueous extract (pVg AE), containing the thermostable Kv1.3-blocking peptide BmKK2, significantly ameliorated diet-induced NASH in mice. Treatment reduced hepatic steatosis, inflammation, and fibrosis by inhibiting Kv1.3-mediated macrophage activation and suppressing the downstream NF-κB/TNF-α signaling axis.^42^ This study provides the first direct *in vivo* evidence linking a scorpion venom-derived peptide to the attenuation of metabolic liver disease.

In addition to this direct evidence, the broader anti-inflammatory mechanisms of isolated venom peptide fractions suggest additional hepatoprotective potential. Linear NDBPs such as ToAP2 have demonstrated a capacity to accelerate the clearance of intracellular pathogens and reduce the overarching structural leukocyte burden and inflammatory cell infiltration within hepatic tissues.^43^ Based on the demonstrated anti-inflammatory pathways of these peptides, it is hypothesized that they may also modulate hepatic fibrogenesis through indirect suppression of Kupffer cell hyperactivation. Under pathological conditions, Kupffer cell-derived TGF-β1 binds to receptors on quiescent HSCs, driving their phenotypical transformation into proliferative, contractile, α-smooth muscle actin (α-SMA)-positive myofibroblasts.^41^^,^^44^ These activated myofibroblasts are the primary drivers of excessive extracellular matrix (ECM) synthesis, resulting in abnormal collagen deposition that distorts hepatic architecture and impairs portal blood flow.^45^ By placing a molecular brake on macrophage hyperactivation via NF-κB and MAPK pathway inhibition (as demonstrated for BmKK2^20^^,^^42^ in nonhepatic models), scorpion venom peptides may indirectly prevent HSC activation, lower systemic matrix metalloproteinase imbalances, and reduce localized collagen accumulation. However, direct validation of this proposed antifibrotic mechanism in hepatic tissue models remains an important direction for future investigation.^40^^,^^41^^,^^44^

### 5.2 Antiviral peptides in chronic viral hepatitis

Alongside nonviral applications, managing persistent viral hepatitis infections remains a core therapeutic strategy to prevent long-term hepatocellular carcinoma. Viral hepatitis strains exploit specific host cell surface structures to gain entry and subvert cellular machinery, making entry inhibition a high-priority drug target.

The peptide Smp76, a 76-amino-acid DBP isolated from Scorpio maurus palmatus, contains six tightly regulated cysteine residues that fold into a rigid cysteine-stabilized α/β (CSα/β) structural motif. Smp76 exhibits potent antiviral activity against dangerous positive-sense RNA flaviviruses, including HCV, Dengue Virus (DENV), and Zika Virus (ZIKV). Mechanistically, Smp76 directly targets and inactivates extracellular viral particles, permanently disrupting their structural integrity and preventing host cell viral entry.^46^ Furthermore, recombinant Smp76 has been shown to upregulate the expression of IFN-β by activating interferon regulatory transcription factor 3 (IRF3) phosphorylation, thereby enhancing the type-I interferon response and suppressing established viral infection through an immunomodulatory mechanism distinct from its direct virucidal activity.^47^

The linear peptide Eval418 (derived from Euscorpiops validus) targets early viral-host cell membrane fusion configurations. While the native peptide sequence displays limited cellular uptake, the strategic computational insertion of histidine residues yields the optimized analog Eval418-FH5, which exhibits superior amphipathic α-helicity, enhanced metabolic stability, and minimal cytotoxicity during active viral neutralization against Herpes Simplex Virus type 1 (HSV-1).^48^

Furthermore, the peptide Ctry2459 (from Chaerilus tryznai) effectively neutralizes circulating HCV particles, while its highly cationic, histidine-rich derivatives (Ctry2459-H2 and Ctry2459-H3) significantly lower the total intracellular accumulation of viral proteins within infected cell lines.^49^ For Hepatitis B virus (HBV) therapeutic frameworks, the cationic peptide BmKDfsin4 directly interferes with viral gene expression to suppress active viral replication, exhibiting potent dose-dependent inhibition of HBV DNA (IC_50_ = 1.26 μM), HBeAg (IC_50_ = 3.95 μM), and HBsAg (IC_50_ = 2.28 μM) with low cytotoxicity.^50^ Similarly, Mucroporin-M1 acts as a structural host defense template that effectively down-regulates the essential liver-specific host transcription factor, hepatocyte nuclear factor 4 Alpha (HNF4α). Because HBV replication is completely dependent on host HNF4α binding to viral core promoters, this peptide-mediated downregulation effectively starves the virus of its required replication machinery, reducing the long-term inflammatory and metabolic burden on the host liver.^51^ These hepatic applications are summarized further in Table 2.

**Table 2:** Scorpion venom-derived peptides as therapeutic agents against hepatic disorders.

Peptide	Source	Characteristics	Target Virus	Mechanism of Action	Ref
Ctry2459	Chaerilus tryznai/C. tricostatus	13 amino acid residues; helical, amphipathic	HCV	Destabilizes viral structural integrity, reducing initiation of HCV infection	^49^
Ctry2459-H2, H3	Derived from Ctry2459	Histidine-rich Ctry2459 peptide	HCV	Reduce intracellular viral levels	^49^
Eval418	Euscorpiops validus	12–14 amino acids; alpha-helix structures	HSV-1	Inhibition by disruption of initial steps of infection	^33^
BmKDfsin4	Buthus martensii Karsch	Small cationic peptides; 34–51 amino acid residues with six cysteines	HBV	Inhibitory activity against HBV replication (IC_50_: DNA 1.26 μM, HBeAg 3.95 μM, HBsAg 2.28 μM)	^50^
Mucroporin-M1	Lychas mucronatus	Cationic host defense peptide	HBV	Inhibition of viral replication by decreasing expression of important HBV replication factors (HNF4α)	^51^
Hp1036	Heterometrus petersii	Amphipathic alpha-helical peptide	HSV-1	Inhibition of cell entry by blocking viral-host membrane fusion	^52^
Hp1239	Heterometrus petersii	Amphipathic alpha-helical peptide	HSV-1	Inhibition of cell entry by blocking viral-host membrane fusion	^52^
Hp1090	Heterometrus petersii	Amphipathic alpha-helical peptide	HCV	Inhibition of viral replication	^52^
rEv37	Derived from Euscorpiops validus	78 amino acid residues; scorpine type peptide with CSalpha/beta motif	DENV-2, HCV, ZIKV, HSV-1	Inhibit DENV-2 infection and suppress HCV and ZIKV infections	^53^
Smp76	Scorpio maurus palmatus	76 amino acids with six cysteine residues; cysteine-stabilized alpha/beta fold	DENV, HCV, ZIKV	Inhibits the ability of HCV virus to infect host cells; upregulates IFN-β via IRF3 phosphorylation	^46^^,^^47^
BmKK2/pVg AE	Buthus martensii Karsch	Thermostable Kv1.3-blocking DBP	N/A (NASH)	Ameliorates diet-induced NASH by attenuating Kv1.3-mediated macrophage activation; suppresses NF-kB/TNF-α signaling	^42^

## 6 Challenges in drug discovery

Scorpion peptides show significant potential in therapeutics, particularly in targeting ion channels and receptors. However, drug discovery using these peptides faces several challenges. These include the complexity of separating specific bioactive peptides from scorpion venom. Scorpion peptides are inherently unstable due to enzymatic degradation. The inherent instability of scorpion peptides necessitates the development of advanced delivery systems to overcome enzymatic degradation and poor systemic absorption. Furthermore, peptides can trigger an immune response or have off-target effects. Mass production of scorpion peptides is technically difficult and expensive, and regulatory limitations add complexity. Ethical issues related to the use of animal peptides and challenges in identifying targets further complicate development. Despite these challenges, ongoing advancements in scorpion peptide synthesis, delivery, and stability are driving continued efforts to develop scorpion peptides into effective drugs.^54^^,^^55^

Translating bioactive scorpion venom peptides into clinical candidates demands considerably more than preclinical activity data alone. Structural characterization remains a persistent bottleneck, as a large proportion of venom peptides still lack experimentally resolved 3D conformations, which limits the depth and reliability of any computational analyses applied to them.^56^ Methods such as molecular docking and simulation-based target engagement studies have been used to good effect in other toxin families; yet their application to scorpion venom peptides is still at an early stage, constrained largely by the absence of validated receptor models for most relevant targets.^57^ Broader pharmacological challenges add further layers of difficulty: short plasma half-lives arising from protease activity, poor oral bioavailability, and immunogenic potential each require independent formulation and chemical engineering solutions for every candidate considered.^58^ Addressing these structural and pharmacokinetic gaps in a coordinated and systematic manner is a prerequisite for meaningful progress from venom biology toward viable drug candidates.

## 7 Future directions

Translating scorpion venom peptides into clinically viable therapeutics will require coordinated advances across several disciplines. Three areas appear particularly promising for accelerating progress in the coming years.

First, artificial intelligence and machine learning are increasingly applied to venom-based drug discovery. Recent work has demonstrated high throughput molecular display platforms capable of systematically screening the animal metavenome to identify novel therapeutic peptide candidates with defined target specificities, enabling the generation of vast functional libraries from natural venom scaffolds.^59^ Similarly, bio-informatics approaches for the classification and functional prediction of venom peptides, including deep learning models for conotoxin superfamily assignment and disulfide connectivity prediction, have established frameworks that are increasingly applicable to scorpion toxin engineering.^60^ These computational approaches may substantially reduce the time and cost associated with traditional screening-based drug discovery.

Second, clinical translation of venom-derived peptides has begun to yield tangible outcomes beyond preclinical models. Chlorotoxin (CLTX), a 36-amino-acid peptide originally isolated from the deathstalker scorpion Leiurus quinquestriatus, has been incorporated into chimeric antigen receptor (CAR) T cell constructs for the treatment of recurrent glioblastoma. A Phase I clinical trial (NCT04214392) demonstrated the feasibility and safety of intracavitary CLTX-CAR T cell administration, with three of four patients achieving stable disease and no dose limiting toxicities observed.^61^ While not directly hepatic in application, this trial validates the broader principle that scorpion venom peptides can serve as functional targeting moieties in advanced therapeutic platforms, a concept that could be extended to liver-tropic delivery systems in the future.

Third, advances in nanoformulation and targeted delivery systems offer solutions to the pharmacokinetic limitations that have historically impeded venom peptide therapeutics. Biodegradable nanosystems (including liposomes, polymeric nanoparticles, and lipid nanoparticles) have demonstrated the capacity to protect peptide payloads from proteolytic degradation, extend systemic half-lives, and enable tissue-specific accumulation.^62^^,^^63^ For hepatic applications specifically, nanoparticle platforms functionalized with liver-targeting ligands can preferentially deliver therapeutic cargo to hepatocytes, Kupffer cells, or hepatic stellate cells, thereby enhancing local bioavailability while minimizing systemic toxicity. Integrating scorpion venom peptides such as BmKK2 or ToAP3 into such targeted nanocarrier systems is a practical next step for achieving clinically meaningful hepatic outcomes.^26^^,^^63^

Together, progress in AI-driven peptide engineering, validated clinical approaches, and advanced delivery technologies is advancing scorpion venom therapeutics toward practical application. While substantial work remains, particularly in generating direct hepatic efficacy data for specific peptide candidates, the tools for rational drug development from this natural product source are now in place.^62^^,^^64^

## 8 Conclusion

Venomous animals have a long history of evolution and cover a wide range of species, among which scorpion venom is a rich source of bioactive molecules, such as peptides, proteins, and enzymes with significant pharmacological activities. With the advent of advanced structural biology tools, including cryo-EM and AI-based protein fold prediction, a rich source of peptides that interact specifically and with high affinity with human proteins can now be identified and engineered with high precision. Antiinflammatory effects induced by peptides such as ToAP3, ToAP4, Stigmurin, TsAP-2, Ts14, BotAF, Sc20, and St20 include altered proinflammatory cytokine levels and leukocyte migratory properties as well as inhibition of inflammation-related signaling pathways. Smp76, a scorpion-type natural antiviral peptide, prevents hepatitis C and other flaviviruses from infecting host cells by inactivating extracellular infectious particles without affecting viral replication, while also enhancing the host type-I interferon response.^30^^,^^31^ Recent preclinical evidence has demonstrated that scorpion venom-derived Kv1.3-blocking peptides can directly ameliorate metabolic liver disease in animal models,^26^ bridging the gap between general anti-inflammatory activity and hepatic-specific therapeutic outcomes.

This may improve our understanding of each interaction and lead to the development of effective drugs targeted to specific protein functions. Future research integrating machine learning-driven peptide design, advanced nanodelivery systems, and rigorous hepatic disease models will be essential to advance the most promising candidates toward clinical evaluation. With modern computational, structural, and pharmacological tools now available, there is a growing opportunity to develop scorpion venom peptides into targeted treatments for inflammatory and hepatic disorders.

## CRediT authorship contribution statement

**Jwal Doctor:** Writing – original draft, Writing – review & editing. **Zeal Parikh:** Writing – review & editing. **Advaita Chauhan:** Writing – review & editing. **Anirudh Raina:** Writing – review & editing.

## Informed consent

Not applicable.

## Ethics statement

Not applicable.

## Animal treatment

Not applicable.

## Organ donation

Not applicable.

## Declaration of generative AI and AI-assisted technologies in the writing process

No AI tools were used in the preparation of this article.

## Funding

The authors received no specific funding for this work.

## Data Availability Statement

Not applicable.

## Declaration of competing interests

The authors declare that there are no conflicts of interest regarding the publication of this manuscript. The research was conducted in the absence of any commercial or financial relationships that could be construed as a potential conflict of interest. The authors alone are responsible for the content and writing of this paper.
